# The potential use of physical resilience to predict healthy aging

**DOI:** 10.1080/20010001.2017.1403844

**Published:** 2017-11-21

**Authors:** Anna Schorr, Christy Carter, Warren Ladiges

**Affiliations:** ^a^ Department of Comparative Medicine, School of Medicine, University of Washington, Seattle, WA, USA; ^b^ Department of Aging and Geriatric Research, Institute on Aging, University of Florida, Gainesville, FL, USA

**Keywords:** Resilience, physical stress, healthy aging, biological age, mouse model of aging

## Abstract

Physical resilience is the ability of an organism to respond to stressors that acutely disrupt normal physiological homeostasis. By definition, resilience decreases with increasing age, while frailty, defined as a decline in tissue function, increases with increasing age. Assessment of resilience could therefore be an informative early paradigm to predict healthy aging compared to frailty, which measures late life dysfunction. Parameters for resilience in the laboratory mouse are not yet well defined, and no single standardized stress test exists. Since aging involves multiple genetic pathways, integrative responses involving multiple tissues, organs, and activities need to be measured to reveal the overall resilience status, suggesting a battery of stress tests, rather than a single all-encompassing one, would be most informative. Three simple, reliable, and inexpensive stressors are described in this review that could be used as a panel to determine levels of resilience. Brief cold water immersion allows a recovery time to normothermia as an indicator of resilience to hypothermia, i.e. the quicker the return to normal body temperature, the more robust the resilience. Sleep deprivation (SD) impairs remote memory in aged mice, and has detrimental effects on glucose metabolism. Cyclophosphamide (CYP) targets white blood cells, especially myeloid cells resulting in neutropenia with a rebound neutrophilia in an age-dependent manner. Thus a strong neutrophilic response indicates resilience. In conclusion, resilience promises to be an especially useful measurement of biological age, i.e. how fast a particular organ or tissue ages. The three stressors, cold, SD, and CYP, are applicable to human medicine and aging because they represent clinically relevant stress conditions that have effects in an age-dependent manner. They are thus an attractive perturbation for resilience testing in mice to measure the effectiveness of interventions that target basic aging processes.

## What is physical resilience?

Physical resilience is the ability of an organism to respond to physical stress, specifically, stress that acutely disrupts normal physiological homeostasis. It is the ability to quickly resolve these unexpected or unusual environmental, medical or clinical challenges that should be relevant to a better understanding of the underlying health status of the animal. By definition, resilience would be expected to decrease with increasing age, while frailty, defined as a decline in tissue function and measured by parameters such as walking speed, gait, and grip strength, increases with increasing age. The loss of resilience occurs earlier in life and so may be a causative factor in the development of frailty [1]. Therefore, assessment of resilience could be a highly informative early paradigm to predict absence of biological dysfunction, i.e. healthy aging, compared to frailty, which only measures late life dysfunction. Resilience promises to be an especially useful measurement of biological age, i.e. how fast a particular organ or tissue ages, which has applications in the mouse as a response endpoint for testing anti-aging therapeutics [2].

## Measuring physical resilience

Unfortunately, parameters for resilience in the mouse are not well defined, and no single standardized stress test exists. Because aging is a multifactorial process, integrative responses involving multiple tissues, organs, and activities need to be measured to reveal the overall resilience status. Therefore, a panel of stress tests, rather than a single all-encompassing one, might be more informative. An ideal battery should have enough dynamic range in the response to allow characterization of an individual in easily distinguishable groups as being resilient or non-resilient [3]. Each test should also be simple, reliable, and inexpensive so the panel can easily be duplicated by many different groups. As a panel, three stressors, cold, sleep deprivation (SD), and the chemotherapeutic drug cyclophosphamide (CYP), fit these criteria. The mechanisms of response to cold are multifactorial. SD is a risk factor for insulin resistance and diabetes, memory loss, heart disease, and cancer. CYP targets several different systems but most specifically cells of lymphoid and neutrophilic lineage.

These stressors are also relevant to human medicine and aging. For example, humans can develop intolerance to cold environmental temperatures with increased sensitivity to hypothermia with increasing age [4]. SD is a major health concern in developed countries and is associated with increasing age [5]. Normal aging produces sleep disturbances including sleep fragmentation and sleep loss in humans. CYP is a representative chemotherapeutic agent used extensively in patients for a variety of conditions including cancer and rheumatoid arthritis. Short-term side effects are more severe with increasing age, and intermediate and long-term effects are associated with a general accelerated aging-like state [6].

### Water immersion

Mice partially submersed in a water bath at 22°C for a brief period of time, e.g. 10 min, develop hypothermia. Hypothermia is often defined as any body temperature below 35.0°C (95.0°F) [7,8]. The basis of the assay is to measure body temperature using an implanted digital transponder while the mouse is submersed in a water bath. The mouse is removed either before body temperature drops below 32° or after a specified time, usually 10 min, quickly wiped dry with a towel, placed in a thermos-neutral (30°C) recovery unit, and monitored for how long it takes for body temperature to return to 37°C, or within the limits of normal baseline body temperature. Using this method, mild hypothermia is defined as 32–35°C in an animal that is alert and actively shivering. Body temperature recovery time increases with increasing age in several strains of mice [Ladiges, unpublished observations]. Therefore, the time required to return to normothermia is an indicator of resilience to hypothermia, i.e. the quicker the return, the more robust the resilience.

### Sleep deprivation

The elderly are less tolerant to SD. It has been shown that SD impairs remote memory in aged mice, with changes in gene expression in the hippocampus [9,10]. SD also has detrimental effects on glucose metabolism outside of the brain in peripheral organs. In a chronic SD experiment, young mice were found to be sensitized to insulin and have improved glycaemic control, whereas aged animals became hyperglycaemic and failed to maintain appropriate plasma insulin concentrations [11]. It has also been shown that SD causes memory lapses and hyperglycaemia in old mice. Therefore, decreased or absence of memory lapse as defined by decreased radial maze escape times, and resistance to hyperglycaemia can be used as measures of resilience to SD.

This resilience test requires the integration of a memory test, for example the radial water tread maze, which detects changes in hippocampal function [12–14]. In this task, mice are introduced into an approximately 30 in. circular galvanized enclosure with 0.5 in. of pre-warmed water at the bottom and nine holes in the sides at regular intervals. One of the holes leads to a dark escape ‘safe box’. The three metrics used are latency to escape, the number of errors, and the distance travelled to reach the escape hole. The animals are given three trials per training day, and the testing period can be run across successive days to test long-term memory acquisition. Mice are kept awake on the morning of day 5 testing. Mice are maintained in an awake state starting at the beginning of the light cycle, for 6 h, or 3 h depending on the cohort. The process entails gently tapping on each cage, and if needed, gentle handling and/or brushing the hairs on the back of each mouse with an extra-fine bristle brush. This simple procedure has been validated to maintain an awake state in mice [15].

### Neutrophilic rebound response to CYP

CYP is an alkylating chemotherapeutic agent with immunosuppressive activity. It was introduced in 1958 and for almost 60 years has been considered the backbone of chemotherapeutic regimens in lymphoproliferative diseases as well as a wide range of solid tumours [6]. It is also an integral part of the conditioning regimen commonly applied in myelo-ablative allogeneic stem cell transplantation [16]. The main effect of CYP is due to its metabolite phosphoramide mustard. Administering CYP in high doses is cytotoxic, due to its activity as an alkylating agent, which leads to inhibition of DNA replication and apoptosis of both tumour and myeloid and lymphoid cells. This cytotoxic effect is observed in actively replicating as well as resting, non-replicating cells. Therefore, CYP has gained attraction as an immunosuppressive treatment in a number of autoimmune disorders, such as rheumatoid arthritis, vasculitis, and systemic lupus erythematosus, and also for chronic graft-versus-host disease in recipients of allogeneic stem cell transplants [17].

Rebound neutrophilia is used to assess sensitivity to CYP because of its preferential targeting of myeloid cells. The procedure consists of administering a single intraperitoneal dose of CYP at 150 mg/kg to mice, and then collecting a drop of tail vein blood on days 0, 3, 8, 10, and 12 to determine blood neutrophil count. The neutropenic nadir will consistently occur on day 3, and the neutrophilic nadir should occur between days 8 and 12, but there will be strain as well as gender variation [18; personal experience]. The strength of the rebound neutrophilia occurs in an age-dependent manner, and thus can be used to assess resilience, i.e. a strong neutrophilic response indicates a high level of resilience to respond to this type of chemotherapeutic stress.

## Validating physical resilience stressors

Before the stressor panel can be used for resilience testing in mice, it needs to be validated with an established measure of healthy aging. The Geropathology Grading Platform (GGP) has been aligned with biological age in mice [19], and can be used as a tool to validate resilience testing.

The GGP is based on a standardized set of guidelines developed to detect the presence or absence of low impact histopathological lesions and to determine the level of severity of high impact lesions in organs from aged mice [20]. It generates a numerical score for each age-related lesion in an organ, summed for total lesions, and averaged over multiple mice to obtain composite lesion score (CLS). Studies show that the platform generates CLS that increase with the age of mice in an organ-dependent and strain-independent manner. In addition, CLS are sensitive enough to detect changes elicited by interventions that extend mouse lifespan, and correlate well with other measures of aging such as cardiac aging [19]. Left ventricular mass index is a measure of chronic progressive heart disease and dysfunction. CLS for heart are an age-sensitive indicator of increasing cardiac dysfunction with increasing age. A second example is the age-dependent increase in carpal joint lesions in association with a decrease in grip strength of the front paw [21]. Thus the GGP is a novel tool to measure biological aging, and help validate resilience as a sensitive indicator of healthy aging compared to any type of frailty index.

In addition to the GGP, a serum biomarker of biological age would help validate resilience testing and also have tremendous translational value [22]. For example, MCP-1/CCL2, a chemokine responsible for recruiting monocytes that also is secreted by senescent cells [23], has been suggested as a biomarker of biological age [22]. Circulating MCP-1 levels increase in an age-dependent manner in wild-type (WT) mice [22]. This age-dependent increase is accelerated in *Ercc1^−/^* ^Δ^ and *Bubr1^H/H^* mouse models of progeria [24]. Genetic and pharmacologic interventions that slow aging of *Ercc1^−/^* ^Δ^ mice attenuate increases in circulating MCP-1. Likewise, rapamycin, which extends lifespan of mice, significantly reduces serum MCP-1 levels in older WT mice. Finally, in older people with cardiovascular disease, MCP-1 levels are significantly higher in frail individuals compared to non-frail peers. It is important to note that serum MCP-1 levels are readily translated to human studies. Currently there are more than 175 clinical trials listed on *ClinicTrials.gov* that incorporate measurement of MCP-1, typically as a marker of inflammation. Therefore, MCP-1 is an excellent surrogate for measuring mammalian biological age that correlates with resilience and response to interventions that extend healthy aging. Experiments with immunosuppressive agents would have to be appropriately controlled.

## Development of a physical resilience scoring platform

In order to implement use of a resilience stressor panel for preclinical aging studies, some type of resilience scoring system needs to be developed. A hypothetical scoring platform is shown in Table 1. Each stressor is graded with a numerical score from 0 to 3 depending on the decreasing ability to respond, i.e. the higher the score, the lesser the resilience. The scores are summarized for each mouse so that a score of 0 would be highly resilient, and a score of 12 would indicate lack of resilience. Scores from each mouse in a cohort would be averaged so that a composite resilience score would be generated, allowing correlation with CLS generated by the GGP, as well as with MCP-1 levels. This scoring system can be used to align with biological age, and also provide the ability to observe the influence of one stressor on the response to another within the panel. As the stressor tests are evaluated in forthcoming experiments, it may be that one or more will prove not to be informative, and deleted from the panel, and new ones added. The scoring platform would accordingly be reconfigured.

**Table 1.** The development of a scoring platform for resilience stressor testing is based on the ability to recover from the panel’s acute stress responses.

Stressor	Measurement	Score	Descriptor
Cold water immersion	Recovery time to 37°	0	<10 min
		1	10–15 min
		2	16–20 min
		3	>20 min
Sleep deprivation	Radial maze escape time	0	<30 s
		1	30–90 s
		2	91–200 s
		3	>200 s
Sleep deprivation	Blood glucose	0	130–150 mg/dL
		1	151–175 mg/dL
		2	175–210 mg/dL
		3	>210 mg/dL
Cyclophosphamide	Rebound neutrophil count	0	>8000 cells/µL
		1	8000–6000 cells/µL
		2	6000–4000 cells/µL
		3	4000–2000 cells/µL

Each stressor is graded with a numerical score from 0 to 3 depending on the decreasing ability to recover, i.e. the higher the score, the lesser the resilience.

## Conclusions and future directions

There is a need to establish the stress test panel as a measure of biological age in order to show that resilience is a measure of aging, and that it can be used as an endpoint for anti-aging therapeutic testing. In order to establish resilience testing as a measure of aging, there must be documentation that the stress assays used to assess resilience will generate data in a way that reflects increasing age or reversal of aging. There are several ways to do this. One way is to compare stress-induced data in young, middle, and old age groups of mice. A second way is to compare data in middle-age mice treated with a well-documented anti-aging drug. These studies have not yet been done.

The resilience stressor panel described in this report represents a multisystem approach for preclinical testing of anti-aging therapeutics that could be utilized at an earlier age and more accurately than frailty assessment in the mouse. The panel is ideal because the individual stressors have a combined dynamic range in the response to allow characterization into easily distinguishable levels of resilience. Each test is simple, reliable, and inexpensive so the panel can easily be duplicated by many different groups. The stressors are also relevant to human medicine and aging. The panel therefore has the potential of being an attractive translational perturbation for resilience testing in mice to measure the effectiveness of interventions that target basic aging processes. These stressor tests, either singly or as a panel, could be adapted to humans in the clinic or in the laboratory on primary cells such as myeloid cells or fibroblasts, to approximate resilience to declining dysfunction associated with increasing age.
